# Cost-utility framework to evaluate therapeutic interventions targeting reduction in cerebral infarction among aneurysmal subarachnoid hemorrhage patients

**DOI:** 10.1007/s10143-026-04201-4

**Published:** 2026-03-26

**Authors:** Adnan I. Qureshi, Nived J. Ranjini, Yilun Huang, Hassan Raza, Thomas Sandifer, Jonathan Beall, Christy N. Cassarly, Byron Gajewski, Renee H. Martin, Camilo R. Gomez, Jose I. Suarez

**Affiliations:** 1 Zeenat Qureshi Stroke Institute, Columbia, MO USA; 2 Department of Neurology, University of Missouri, Columbia, MO USA; 3 Department of Statistics, University of Missouri, Columbia, MO USA; 4 Clinical Pharmacy Specialist, University of Missouri, One Hospital Dr., Columbia, MO USA; 5 Department of Public Health Sciences, College of Medicine, Medical University of South Carolina, Charleston, SC USA; 6 Department of Biostatistics & Data Science, University of Kansas, Kansas City, KS USA; 7 Division of Neurosciences Critical Care, Departments of Anesthesiology and Critical Care Medicine, Neurology, and Neurosurgery, The Johns Hopkins University School of Medicine, Baltimore, MD USA

**Keywords:** Cost-utility, Subarachnoid hemorrhage, Quality of Life Years, Cerebral Infarction, Therapeutic intervention

## Abstract

We developed a framework to assess cost-utility of potential therapeutic interventions targeting reduction in cerebral infarction in aneurysmal subarachnoid hemorrhage (aSAH) patients prior to investing in high cost randomized controlled trials. We estimated the cost and Quality-Adjusted Life Years (QALYs) for 100 hypothetical aSAH patients varying the proportion of patients who develop cerebral infarction (35%, 30%, 25%, and 20%) during initial hospitalization. We estimated both cost and QALYs at 1, 5, and 30-year time. We compared the net costs of therapeutic interventions that cost $5,000, $10,000, $15,000, and $20,000 per patient to simulate costs of existing and potential therapeutic interventions. In the base case in which 35% of the 100 aSAH patients develop cerebral infarction, the total cost was $13,777,940, with total QALYs of 56.9 at 1 year. The total cost was lowest for 100 aSAH patients in the scenario where only 20% of them developed cerebral infarction with total cost estimated at $13,012,653 and QALYs of 60.8 at 1 year. A therapeutic intervention that costs $5,000 per patient (for example: enteral nimodipine, cilostazol or IV 25% humanized albumin alone or in various combinations) was cost effective at 1 year with 10% and 15% reduction in cerebral infarction (compared to the base case) and at 5 years with 5%, 10%, and 15% reduction in cerebral infarction based on a health system expense threshold (willingness to pay) of <$50,000 per QALY gained. A therapeutic intervention that costs $15,000 per patient (for example: IV clazosentan) was cost effective at 5 years only with 15% reduction in cerebral infarction under willingness to pay <$100,000 per QALY. We present a cost-utility framework which allows pre-trial assessment based on the cost of a therapeutic intervention and the expected magnitude of reduction in occurrence of cerebral infarction in aSAH patients.

## Introduction

The American Heart Association/American Stroke Association (AHA/ASA) and the Neurocritical Care Society (NCS) guidelines for the management of aneurysmal subarachnoid hemorrhage (aSAH) identify delayed cerebral ischemia (DCI*)*, as a major cause of death and disability in patients with aSAH [1, 2]. Oral nimodipine is the only agent targeting DCI that has been shown to improve neurological outcomes in patients with aSAH (AHA: Class I recommendation; Level of Evidence A; NCS: Strong Recommendation; moderate quality of evidence) [1, 2]. Other agents such as endothelin-1 antagonists, magnesium sulfate, and statins have failed to demonstrate any benefit in phase 3 randomized controlled trials (RCTs) emphasizing the need for new strategies to reduce the death and disability associated with DCI in aSAH patients. There has been a shift focusing on reduction in cerebral infarction as the therapeutic target instead of angiographic vasospasm or symptomatic vasospasm to reduce death or disability. Reduction in angiographic and/or symptomatic vasospasm has not resulted in any decrease in the rate of death or disability in subsequent Phase 3 RCTs [3, 4]. Cerebral infarction, is an endpoint that allows assessment of vasospasm-independent pathways [3, 5, 6] such as autoregulatory failure independent of angiographic vasospasm [7, 8] and is predictive of both qualitative and quantitative components of treatment effect measured by Phase 3 RCT end-points [9–11]. 

There is also emphasis to develop cost effective therapeutic interventions in aSAH patients. The standard approach has been to develop a therapeutic agent, test it through RCTs and assess cost-utility after efficacy has been demonstrated [4, 12]. RCTs of health care interventions are increasingly attempting to tackle issues of cost-utility prior to investing in high cost RCTs [13]. The median capitalized research and development investment to bring a new drug to market was estimated at $985.3 million and the mean investment was estimated at $1,335.9 million in an analysis of products approved by the United States (USA) Food and Drug Administration (FDA) [14]. 

We report a cost-utility framework which allows pre-trial assessment of a new therapeutic agent based on the cost of the therapeutic intervention and expected magnitude of reduction in occurrence in cerebral infarction in aSAH patients.

## Materials and methods

### Occurrence of new cerebral infarction in aSAH patients

We assumed that the base rate of occurrence of new cerebral infarction in aSAH patients during initial hospitalization is 35% based on a previous meta-analysis of 24 RCTs (1,182 of 3,474 placebo treated patients) [15], single center studies (202 of 582 patients and 149 of 423 patients) [16, 17], and results from the Clazosentan to Overcome Neurological Ischemia and Infarct Occurring After Subarachnoid Hemorrhage (CONSCIOUS)-2 trial (134 of 383 placebo patients) [18]. The estimate is lower than the 39% occurrence (56 of 143) reported by Rabinstein et al. [19] and 46% (191 of 413) reported by Ayling et al. [20] and 46% (56 of 126) reported by Wong et al. [21].

### Death or disability in patients with and without cerebral infarction

Table 1 summarizes the distribution of proportion of aSAH patients in each category of modified Rankin scale (mRS) at various time points. We estimated the proportion of aSAH patients in each category of mRS at 3 months according to presence or absence of cerebral infarction during initial hospitalization based on a previous study by Wong et al. [21]. We estimated the proportion of aSAH patients who were classified as functionally independent (mRS 0–2), functionally dependent (mRS 3–5), and dead at 12 months according to presence or absence of cerebral infarction based on a previous study by Rabinstein et al. [22]. We estimated the change in mRS in patients 1 and 5 years post hospitalization based on the study by Lee et al. [23]. We estimated the proportion of patients with functionally independent, functionally dependent, or dead at 2 years, 3 years, and 5 years. The transition between mRS grades plateaus after year 4 [23] and we assumed that after 5 years, the proportion who were classified as functionally independent or functionally dependent does not change. After 5 years, there was no transition expected between patients who were functionally independent or dependent. However, the risk of death was still modeled using transition probabilities from Huhtakangas et al. [24]. We simulated patient disability (mRS) and death using a simple Markov model over 30 years. Since the study by Huhtakangas et al. [24] used Glasgow Outcome Scale, we converted the Glasgow Outcome Scale to mRS based on validated conversion scheme proposed by Gaastra et al. [25].

### Utilization of nursing home

Previous studies have assumed that patients discharged to a skilled nursing facility (SNF) or inpatient rehabilitation (IPR) facility by 3 months would remain in a long-term care setting for the remainder of their lives [26, 27]. However, recent studies involving aSAH patients have shown that patients discharged to nursing homes can eventually leave for home and that discharge from the nursing home is not influenced by occurrence of cerebral infarction during initial hospitalization [28, 29]. We estimated the utilization of nursing homes at 3 months according to mRS grades as described by Shireman et al. [26]. We assumed a total of 70% of patients in nursing homes at 3 months will still be in nursing homes at 1 year according to Greebe et al. [29]. Using transition probabilities, we estimate the proportion of patients who remained in nursing homes at years 2, 3, and 5. We assumed that a total of 61% and 36% of patients in nursing homes at 1 year will still be in nursing homes at 2 and 5 years, respectively. At 5 years we assumed that patients who were discharged to nursing home will still be in nursing home with no further discharges expected [29]. After 5 years, we assumed that the annual rate of continuing in nursing home admission was 13%.

### Cost of acute hospitalization

Table 1 summarizes the various assumptions for cost used in analysis. Costs for aSAH patients who developed or did not develop cerebral infarction during initial hospitalization were derived from Nationwide Inpatient Sample (NIS) in from 2016 to 2021 [30]. The NIS is the largest all-payer inpatient care database in the USA and contains data from a representative sample of approximately 20% of USA hospitals and has been used in previous aSAH studies [31–34]. The NIS data is derived from billing data submitted by hospitals to statewide data organizations across the USA. These inpatient data include clinical and resource use information typically available from discharge abstracts to provide national estimates of health care utilization, access, charges, quality, and outcomes.

### Cost of post hospitalization care

The estimate was made based on two steps:

1. Cost for post hospitalization according to disability:

The one-year post hospitalization cost was assigned using the estimates provided by Shireman et al. [26] for each mRS grade. The estimates provided by Shireman et al. were derived from acute ischemic stroke patients but a previous study had not identified any differences between ischemic stroke patients and aSAH patients in post hospitalization cost according to disability [35]. Shireman et al. [26] used Medicare payments from inpatient and outpatient claims extending from day 91 after the index hospitalization until death or censoring that were summed and divided by the period of observation to annual averages for patients who were functionally independent or dependent in 2014. After the first year, the cost was calculated at three time points between 1-year and 5-year (2-year, 3-year, and 5-year) to account for change in status of patients transitioning from functionally dependent into independent status and those who died based on the study by Lee et al. [23]. The cumulative total was multiplied by 1.33 to get an overall 4 year cost. This was added to the first-year cost to get the aggregate 5-year cost. The fine granularity in annual cost estimates was necessary because the first year was disproportionately higher to other years and the annual cost between year 1 and year 5 was not stable due to transition in mRS grades.

2. Cost for nursing home care post discharge:

The cost of nursing home was exclusive of the cost for post hospitalization care that was seen in all aSAH patients. Nursing home costs were estimated using Genworth’s 2025 Cost of Care Survey and applied based on functional status at 3 months post-discharge [36, 37]. While patients who were functionally dependent (mRS 3–5) were the primary users of long-term nursing home care, a small proportion of functionally independent patients (mRS 0–2) also incurred short-term nursing home costs, typically for rehabilitation or transitional care. These costs were lower and adjusted proportionally using observed utilization rates.

To reflect the decreasing likelihood of nursing home use over time, we applied a 0.85 adjustment factor to the first-year cost estimates from Genworth to account for patient discharges between 3 months and 1 year. The cumulative nursing home cost over years 1–5 was then multiplied by 1.33 to estimate a 4-year cost, consistent with the approach used for post-hospital medical care in Step 1.

### Cost after 5 years

The annual cost for patients who were functionally independent or functionally dependent was assumed to not change throughout the life expectancy and we used the estimates used by by Shireman et al. [26].

### Cost calculation

The estimated mean net cost for 100 patients with aSAH was the sum of the following costs: initial hospitalization (all 100 patients), ongoing disability (all alive patients), and nursing home utilization (only those residing in nursing home). All cost were adjusted to 2025 dollars using the Consumer Pricing Index (CPI) [38]. 

### Quality of adjusted life years at 1-year, 5-year and 30-year

The health benefit of each simulated treatment cost ($5,000, $10,000, $15,000, $20,000) was expressed in QALYs and as reported in previous studies, assigned the following estimates: (a) Functionally independent = 0.74 QALYs; (b) Functionally dependent = 0.38 QALYs; and death = 0 QALYs [39–42]. The QALYs were estimated at two additional time points for 100 aSAH patients, at 5 years and at 30 years, by using the proportion of patients in each category (functionally independent, functionally dependent or dead) at those two time points. The same QALYs of 0.74, 0.38, and 0 were for patients categorized as functionally independent, functionally dependent or dead [39–42]. The QALYs reflect the sum total of QALY for the cohort of 100 patients at year 1 post-aSAH, year 5 post-aSAH, and year 30 post-aSAH.

### Simulation scenarios

We modeled a hypothetical cohort of 100 aneurysmal subarachnoid hemorrhage (aSAH) patients, using a 35% cerebral infarction rate during initial hospitalization as the base case, representing expected outcomes in the absence of additional intervention. We subsequently simulated the 30-year functional status across range of assumptions in hypothetical cohorts of 100 aSAH patients in whom 30%, 25%, and 20% of the patients developed cerebral infarction during initial hospitalization to estimate costs and QALYs.

### Cost-utility of therapeutic interventions

To evaluate the cost-utility of hypothetical interventions that reduce the occurrence of cerebral infarction, we compared total costs and QALYs across simulated cohorts. The difference in cost between the base case (35% cerebral infarction rate) and each hypothetical scenario (30%, 25%, and 20% cerebral infarction rates) was calculated at 1-, 5-, and 30-years. Similarly, the difference in QALYs between the base case and each intervention scenario was used to estimate the incremental QALYs gained. These values were then used to compute the incremental cost-utility ratio (ICER) for hypothetical therapeutic interventions that cost $5,000, $10,000, $15,000, and $20,000, expressed as cost per additional QALY gained with reduction in cerebral infarction incidence, and calculated as:

Cost of therapeutic intervention (e.g. $5,000 per patient) for 100 aSAH patients-Cost saved by reduction (e.g. 5%) in occurrence of cerebral infarction in 100 aSAH patients/Increase in QALYs per 100 aSAH patients (e.g. with 5% reduction in occurrence of cerebral infarction).

**Fig. 1:**
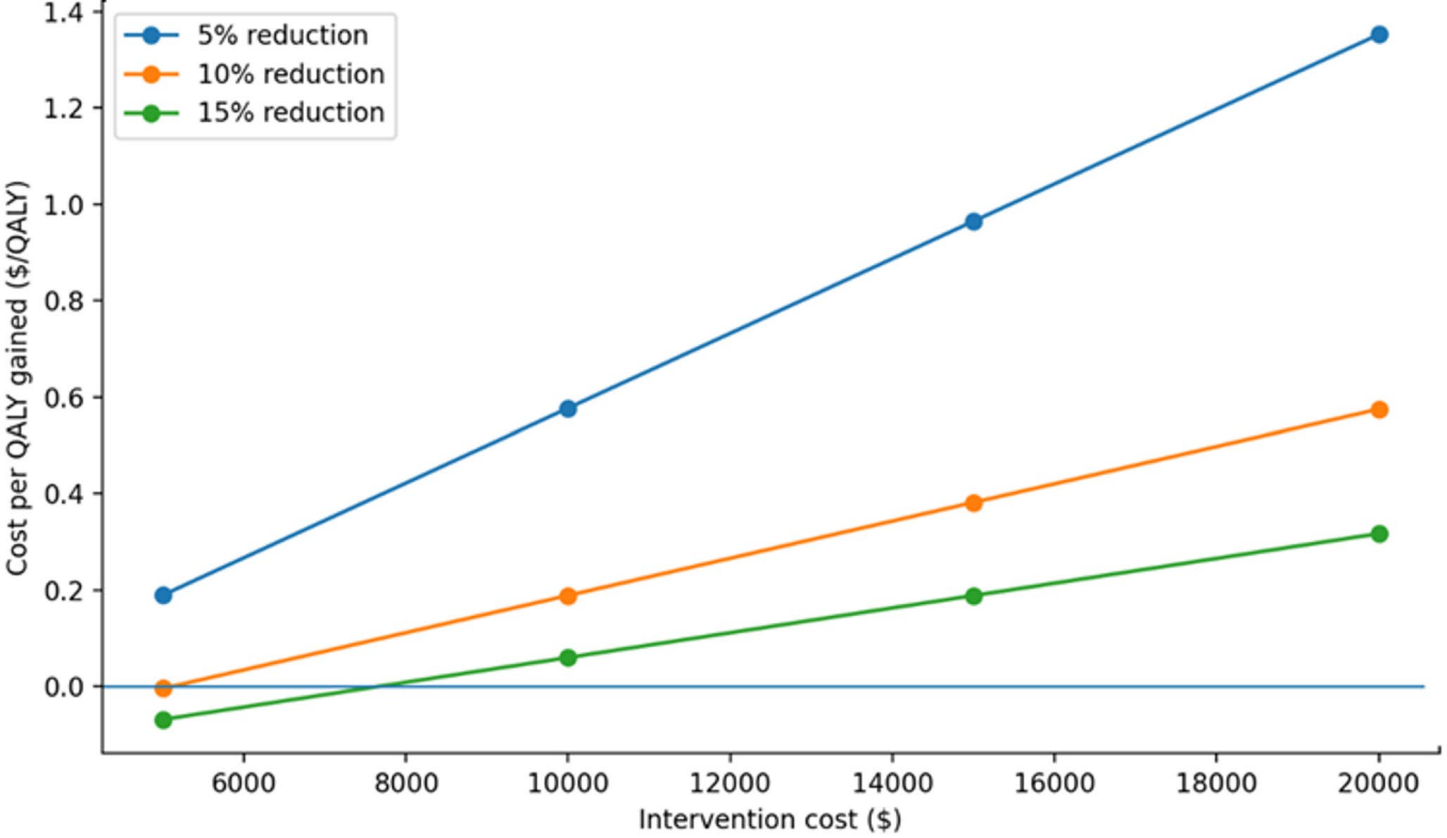
The relationship between ICER and intervention costs at 1 year

**Fig. 2:**
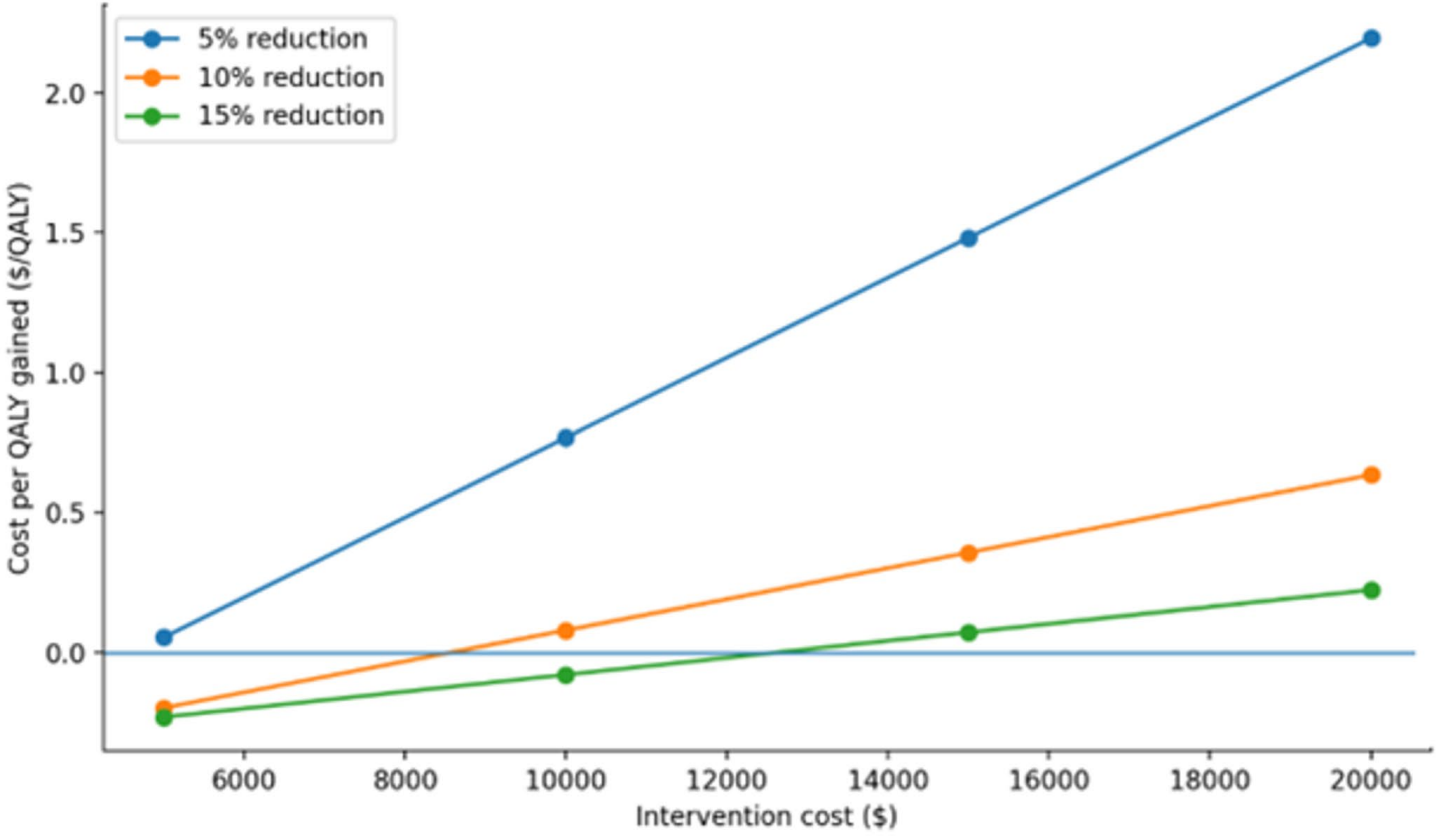
The relationship between ICER and intervention costs over 5 years

**Fig. 3:**
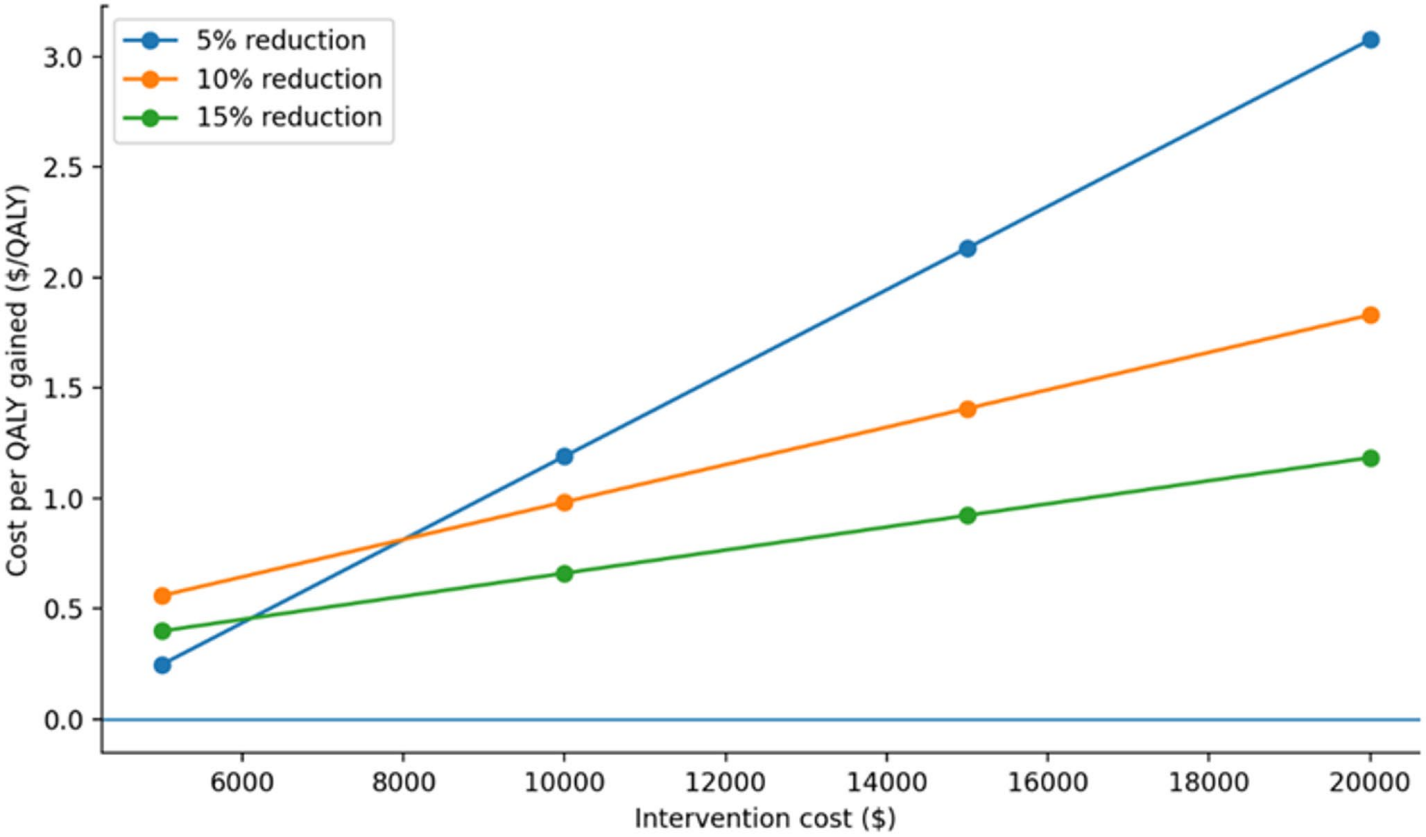
The relationship between ICER and intervention costs over 30 years

The cost per QALY gained was used to assess which scenarios resulted in cost effective therapeutic interventions, based on willingness to pay thresholds of <$50,000, <$100,000 and <$150,000 per additional QALY [39, 43–45]. Cost-utility results were summarized graphically by plotting incremental cost-effectiveness ratios against intervention costs across assumed effect sizes (5%, 10%, and 15% reductions) and analytic time horizons (1-year, 5-year, and 30-year). These figures provide visual comparison of cost-utility patterns and identify scenarios in which interventions exceeded conventional willingness-to-pay thresh­olds [Figures 1, 2 and 3].

### Deterministic sensitivity analysis

A deterministic sensitivity analysis was performed and summarized using a tornado diagram, constructed from the same unit costs, patient distributions, and aggregate outcomes used to generate the 1-year, 5-year, and 30-year cost-utility estimates. The analysis was anchored to the 5-year horizon, as functional outcomes were assumed to plateau beyond 5 years and because most of cost divergence between aSAH patients with and without cerebral infarction occurred during this period. The effect of each key model parameter contributing to incremental cost or QALY differences was estimated under multiple assumptions across plausible ranges while all other inputs were held constant.

### Cost-utility of existing and potential therapeutic interventions

Using our cost-utility estimates, we established whether existing interventions meet the cost-utility thresholds based on estimates of potential effect from a literature review. We included enteral nimodipine which is approved for use in patients with aSAH [46, 47], enteral cilostazol which has been assessed in several RCTs [48, 49], intravenous (IV) 25% humanized albumin that was assessed in a phase 2 clinical trial [50], and IV clazosentan which has been assessed in several RCTs [51] and is currently approved for use in Japan. We also assessed the cost-utility of combination treatment which was identified as an area of potential interest in 2023 AHA/ASA guidelines [52]. We considered the combination of enteral nimodipine, cilostazol and IV 25% humanized albumin that may have therapeutic potential based on preliminary data [53]. 

The costs of medication was determined from National Average Drug Acquisition Cost (NADAC) 2025 [54]. IV 25% humanized albumin was not listed on NADAC and Average Wholesale Price (AWP) was used in place of NADAC. Albumin dose was based on a patient weight of 80 kg; 1.25 g/kg * 80 kg = 100 g of Albumin prescribed; each bag of 25% albumin contains 12.5 g of albumin; 8 bags will be dispensed for each prescribed dose of albumin. IV clazosentan cost was provided in a previous analysis in Japanese market and was converted to USD (July 18th, 2025) [55, 56]. 

## Results

### Effect of reduction in cerebral infarction on 1-Year Cost and QALYs

In a simulated cohort of 100 aSAH patients in which 35% of the patients developed cerebral infarction (“base case”), the total estimated cost for the patients assumed to develop cerebral infarction was $5,982,964 ($170,941.80 per patient) with total QALYs of 14.0. In the 65% of patients simulated not to develop cerebral infarction, the total estimated cost was $7,794,976 ($119,922.40 per patient) with total QALYs of 42.9. Therefore, the total cost for all 100 simulated patients was $13,777,940 with total QALYs of 56.9 at 1 year. Table 2 demonstrates the effect of reducing the assumed proportion of patients who develop cerebral infarction from the base case (35%) to 30% (5% reduction), 25% (10% reduction), and 20% (15% reduction). The total cost was lowest with 15% reduction in proportion of patients who develop cerebral infarction with total cost estimated at $13,012,653 and total QALYs increased to 60.8.

### Effect of reduction in cerebral infarction on 5- year cost and QALYs

At 5 years, the total estimated cost and QALYs in the 35% of the patients who developed cerebral infarction was $9,529,902.30 ($272,282.90 per patient), and 13.5, respectively. The estimated cost in the 65% of the patients who did not develop cerebral infarction was $12,303,601.90 ($189,286.20 per patient) with QALYs of 42.6 at 5- years. The total cost for 100 aSAH patients was $21,833,504.20 with QALYs of 56.1 at 5- years. The total cost was lowest with 15% reduction in proportion of patients who develop cerebral infarction with total cost estimated at $ 20,571,518.30 and QALYs increased to 59.4 (Table 2).

### Effect of reduction in cerebral infarction on 30-year cost and QALYs

The estimated cost and QALYs in the 35% of the patients who developed cerebral infarction was $ 10,112,038 ($288,915.40 per patient) with QALYs of 3.08 at 30 years. The estimated cost and QALYs in the 65% of patients who did not develop cerebral infarction was $28,969,455 ($445,683.90 per patient) with QALYs of 15.2 at 30 years. The total cost for 100 aSAH patients was $60,914,997 with QALYs of 18.3.

### Cost per QALY with hypothetical therapeutic interventions at 1-,5-, and 30-year timepoint

Table 3 presents the cost per QALY gained under various scenarios when cost of therapeutic intervention ranged from $5,000 per patient to $20,000 per patient. The scenarios also assessed cost per QALY gained when therapeutic intervention resulted in 5%, 10%, and 15% reduction in proportion of patients who develop cerebral infarction. A therapeutic intervention that costs $5,000 per patient ($500,000 per 100 aSAH patients) was cost effective at 1- year with 10% and 15% reduction in cerebral infarction and at 5- years with 5%, 10%, and 15% reduction in cerebral infarction under willingness to pay of <$50,000 per QALY gained in simulated cohort of 100 aSAH patients. A therapeutic intervention that costs $10,000 per patient was cost effective at 1- year with 15% reduction in cerebral infarction and at 5- year timepoint with 10%, and 15% reduction in cerebral infarction. A therapeutic intervention that costs $15,000 per patient was cost effective only at 5- year timepoint with 15% reduction in cerebral infarction under willingness to pay of <$100,000 per QALY. A therapeutic intervention that cost $20,000 per patient and resulted in a 10% or 15% reduction in the proportion of patients who developed cerebral infarction was not cost effective at any time point even with the willingness to pay of <$150,000 per QALY [Figures 1, 2 and 3].

**Fig. 4:**
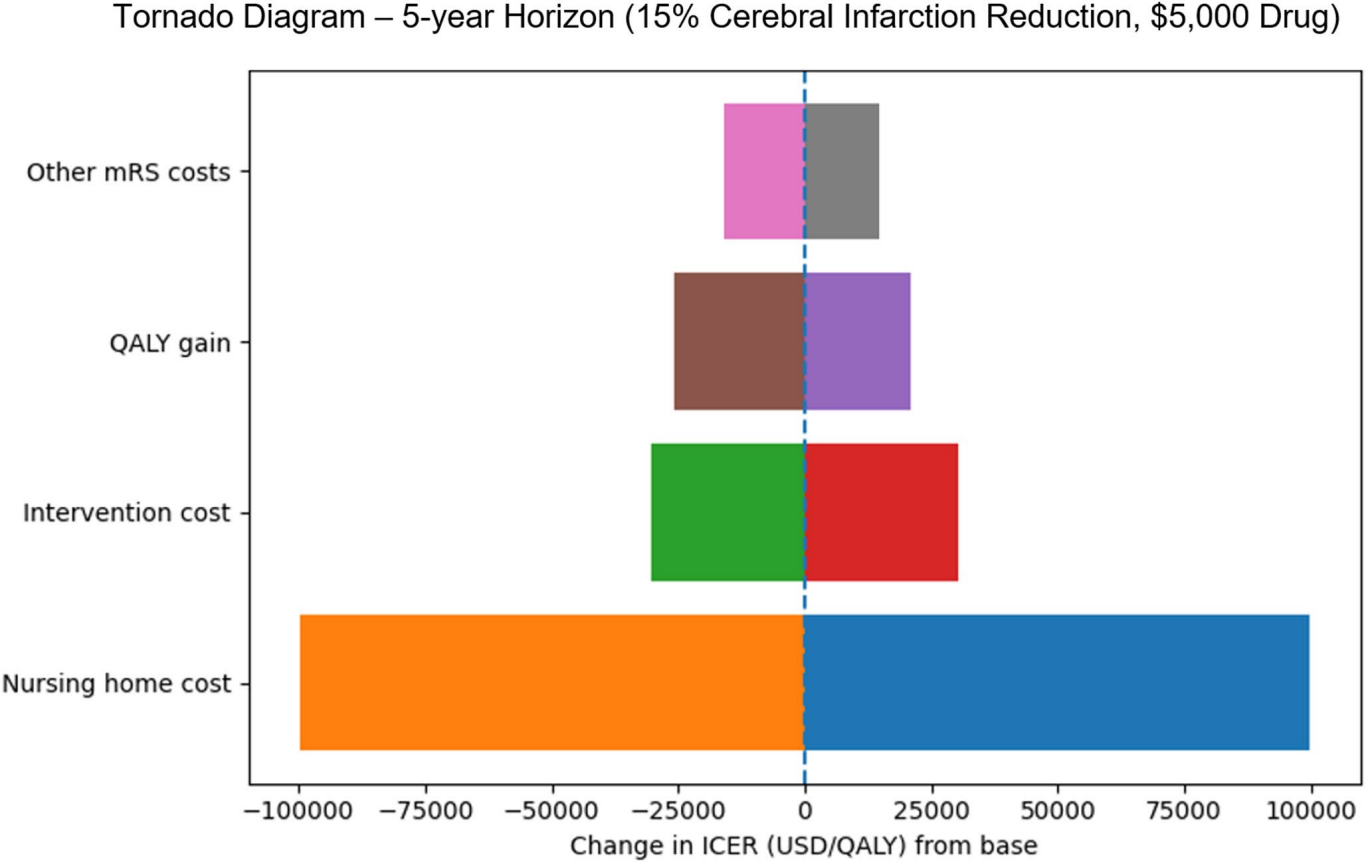
Tornado diagram of one-way sensitivity analysis at the 5-year horizon

The tornado diagram (Fig. 4) summarizes one-way deterministic sensitivity analyses evaluating uncertainty in key cost and utility inputs and demonstrates that the base-case cost-utility conclusions shown in Tables 2 and 3 were robust. Nursing-home costs among functionally dependent patients (mRS 3–5) were the primary drivers of model uncertainty, accounting for the largest variation in the ICER. Varying nursing-home costs by ± 20% resulted in ICER shifts of approximately ±$100,000 per QALY. Intervention cost had a smaller but meaningful influence (±$30,303 per QALY), while variation in QALY gains (± 10%) produced ICER changes ranging from −$25,656 to +$20,991 per QALY. Changes in other long-term medical costs among functionally independent patients (mRS 0–2) had the least impact (±$14,496 per QALY). Across all plausible parameter ranges, the intervention remained cost-saving over the 5-year horizon, confirming the stability of our findings.

### Cost-utility of existing and potential therapeutic interventions

The practical implications of the model are that enteral cilostazol (in 200 mg per day or 300 mg per day), enteral nimodipine, and IV 25% humanized albumin (as a single dose or 7 doses of 1.25 mg/kg mg per day), which cost under $5,000 per patient (see Table 4), would be cost-effective in all the scenarios considered. In contrast, IV clazosentan, which costs approximately $15,000 per patient, would be rarely cost-effective [Table 4].

## Discussion

The cost-utility framework allows pretrial assessment of a new therapeutic agent based on: 1/. cost of the intervention; 2/. magnitude of reduction in poor outcome; 3/. duration of follow up time, and 4/. willingness to pay for additional QALY. For trials of aSAH patients, magnitude of reduction in occurrence of cerebral infarction with a therapeutic intervention can be estimated in Phase 2 trials and, with this estimate, the cost-utility framework described in this paper allows an assessment of cost-utility prior to moving to Phase 3 trials [4]. The assessment under various assumptions is valuable because the payers and society may want to consider a shorter time frame such as 1- or 5- years to determine cost-utility instead of 30- year if the majority of aSAH patients are elderly or have a shorter life expectancy. The willingness to pay may differ between societies based on gross domestic product [57], and certain therapeutic interventions such as IV clazosentan may be cost effective in high income countries but not in low-income countries.

There was only a modest increase in costs between 5 and 30 years among aSAH patients who developed cerebral infarction compared with those without cerebral infarction. The apparent difference in cost accumulation between aSAH patients with and without cerebral infarction reflects differences in long-term survival rather than inconsistencies in cost estimation. Patients with cerebral infarction experience substantially lower survival, resulting in fewer surviving years during which ongoing disability-related and nursing home costs can accrue. Consequently, although their per-patient costs are high in the first 5 years, additional costs between years 5 and 30 increased only modestly because a large proportion of these patients did not survive long enough to incur long-term costs. In contrast, patients without cerebral infarction have higher long-term survival, incurring costs related to disability and nursing home care over many additional years.

There are certain considerations to make prior to interpreting the cost-utility framework. First, we are assuming that the difference in costs and QALYs according to proportion of patients who develop cerebral infarction, e.g. between 35% and 20% is synonymous with 15% reduction in cerebral infarction with a therapeutic intervention. Our model assumes that the effect of cerebral infarction on death or disability is not confounded by other factors such as initial severity of neurological injury or elderly age of patient, which coexists in patients with cerebral infarction. We cannot exclude the possibility that such assumptions can result in overestimation of the QALY benefits of the hypothetical interventions. Previous studies have confirmed that the effect of occurrence of cerebral infarction on death or disability is independent of other prognostic factors but the estimate in multivariate analysis may vary from odds ratio of 2.13 [58] to 4.89 [59]. The cost of initial hospitalization in aSAH patients was higher than those used in previous models [39, 60], but more recent studies have identified a higher cost of hospitalization for patients with aSAH similar to our estimates [30, 61, 62]. Previous models using Medicare based reimbursement [26] have underestimated the cost incurred through nursing homes since Medicare covers the cost for the first 20 days completely, and then covers a daily coinsurance for days 21–100 ($209.50 per day) [63]. Our cost-utility framework does not take into account the indirect cost such as lost productivity due to aSAH or death, and the costs of informal caregiving [64]. We estimated the cost of each therapeutic intervention without accounting for any additional monitoring or imaging or laboratory surveillance that maybe required. All of our cost estimates are subject to inflation and local pricing dynamics. Although we adjusted costs using the CPI and institutional pharmaceutical price schedules, residual misestimation is possible because the CPI is a broad, population-level measure of consumer inflation and may not accurately reflect healthcare-specific cost growth, regional variation, or rapid changes in pharmaceutical pricing [65]. The survival rates and hospital cost data used in the analysis are derived from tertiary and quaternary referral centers, which may preferentially receive more severe and complex aSAH cases; this referral bias may limit generalizability to other hospital settings. Finally, our modeling assumes that functional recovery and annual costs plateau after five years following aSAH, which may oversimplify long-term recovery trajectories and resource utilization in this population.

We acknowledge that there are other models for assessment of cost incurred such as the time-driven activity-based costing method which integrates the cost of each resource used in the process and the quantity of time the patient spends with each resource [66–69]. A micro-costing approach [70] based on direct counting of fixed and variable costs for every input consumed in the treatment of a particular patient may allow more in-depth analysis to measure costs of a service as accurately as possible. A micro-costing approach is effective in areas where savings without compromising care can be made within the broader context of aSAH patient care. We used data from multiple studies to model the cost-utility rather than comparison of actual cost derived from a single trial as used in previous cost-utility models [39, 71–74]. The ideal strategy would be derivation of cost from a prospective database of patients from a single institution where costs is actually calculated and thus avoiding a modeling approach. Although, most of the estimates of the absolute treatment effect and cost-utility ratios do not differ between models and actual trials [75–77]. The cost estimates used in the models were derived over a period of time and cost was adjusted to 2025 using the CPI. The inflation in the CPI is based on out-of-pocket spending by consumers and may not always reflect changes in healthcare expenditures funded by government programs (Medicaid, Medicare Part A) and employer-sponsored insurance.

In conclusion, we present a cost-utility framework which allows pre-trial assessment based on the cost of the therapeutic intervention and magnitude of reduction in occurrence of cerebral infarction in aSAH patients. Although this framework relies on hypothetical treatment effects and prices, it offers a structured way to assess emerging therapies as new evidence on efficacy and costs becomes available. Instead of providing statements like “a drug costing $5,000 would be cost-effective” as a clinically actionable conclusion, the model is a flexible tool in which investigators can insert empiric estimates of cerebral infarction reduction, treatment cost, and downstream disability drawn from ongoing and future trials. Importantly, the framework can also be used as a threshold analysis tool, estimating the magnitude of cerebral infarction reduction that would be required, at a given intervention cost, for a novel therapy to meet prespecified cost-effectiveness criteria and thereby justify further evaluation. As candidate agents such as a combination of enteral cilostazol and IV humanized albumin are developed and validated, the framework can be used to quickly produce comparative cost-effectiveness estimates, guide trial design (e.g., identifying target effect sizes and relevant subgroups), and support early health-economic decision-making by clinicians and policymakers.

**Table 1:** Acute and Post-Hospitalization Costs per Patient Used in Analysis

All patients admitted with aSAH (includes both who died and those who were discharged alive)	
With cerebral infarction	$122,200.96
Without cerebral infarction	$89,997.00
All patients who are alive	
Annual cost for each patient with mRS 0–2	$14,293.80
Annual cost for each patient with mRS 3–5	$15,633.30
Only patients residing in nursing homes (in addition to cost for care according to disability)	
Annual cost for nursing home	$114,665.00

Abbreviations used: aSAH=aneurysmal subarachnoid hemorrhage; mRS= modified RankinScore

**Table 2:** Aggregate Costs and QALY Estimates for 100 Simulated aSAH Patients with Varied Proportion Developing Cerebral Infarction

Aggregate Costs and Overall QALY						
	At 1- year		At 5- year		At 30- year	
Patients with new cerebral infarction (%)	Cost	QALYs at 1 year*	Cost	QALYs at 5 year*	Cost	QALYs at 30 year*
35%	$13,777,940	56.9	$21,833,504	56.1	$60,914,997	18.3
30%	$13,522,844	58.2	$21,371,192	56.8	$60,546,080	18.8
25%	$13,267,749	59.5	$20,976,797	57.9	$61,075,366	19.5
20%	$13,012,653	60.8	$20,571,518.	59.4	$61,177,403	20.2

Abbreviations used: aSAH=aneurysmal subarachnoid hemorrhage; QALY= Quality-Adjusted Life Year.

Symbols used: * The QALYs at time points of 1 year, 5 years, and 30 years. The values are not aggregates of annual values.

**Table 3:** Cost-utility of Therapeutic Interventions Across Time by Reduction in Proportion Patients with New Cerebral Infarction

	At 1- year			Aggregate at 5- year			Aggregate at 30- year		
	30% (5% reduction)	25% (10% reduction)	20% (15% reduction)	30% (5% reduction)	25% (10% reduction)	20% (15% reduction)	30% (5% reduction)	25% (10% reduction)	20% (15% reduction)
Overall cost saved	$255,096	$510,191	$765,287	$462,311	$856,706	$1,261,985	$368,916	-$160,368	-$262,406
Overall QALY increase	1.3	2.6	3.9	0.7	1.8	3.3	0.5	1.2	1.9
Cost per QALY gained with $5,000 intervention	$189,848	-$3,934	-$68,197	$53,840	-$198,170	-$230,904	$247,326	$559,634	$399,165
Cost per QALY gained with $10,000 intervention	$577,445	$189,115	$60,337	$768,126	$79,607	-$79,389	$1,190,723	$983,363	$660,945
Cost per QALY gained with $15,000 intervention	$965,041	$382,165	$188,872	$1,482,411	$357,385	$72,125	$2,134,119	$1,407,092	$922,725
Cost per QALY gained with $20,000 intervention	$1,352,638	$575,215	$317,406	$2,196,697	$635,163	$223,640	$3,077,515	$1,830,821	$1,184,505

Abbreviations used: Quality-Adjusted Life Year.

**Table 4:** Cost of Therapeutic Agents for Aneurysmal Subarachnoid Hemorrhage (aSAH) Management

	Cost per dosing Unit	Cost per Day	Cost for Duration of Therapy
Cilostazol 100 mg tablet (200 mg/dX14 days)	$0.13	$0.26	$3.64
Cilostazol 100 mg tablet (300 mg/d X14 days)	$0.13	$0.39	$5.46
IV 25% humanized albumin (1.25 g/kg dose X1 dose)	$55.87	$446.96	$446.96
IV 25% humanized albumin (1.25 g/kg dose x7 doses)	$55.87	$446.96	$3,128.72
Nimodipine 30 mg capsules (60 mgX4 hours) X 21 days	$1.09	$13.08	$274.68
Cilostazol 100 mg tablet (200 mg/dX14 days) and IV 25% humanized albumin (1.25 g/kg dose X1 dose)	$56.00	$447.22	$450.60
Cilostazol 100 mg tablet (200 mg/dX14 days) and IV 25% humanized albumin (1.25 g/kg dose X1 dose) and nimodipine (240 mg/dX21 days)	$57.09	$460.30	$725.28
Cilostazol 100 mg tablet (300 mg/d X14 days) and IV 25% humanized albumin (1.25 g/kg dose x7 doses)	$56.00	$447.35	$3,134.18
IV Clazosentan	JPY ¥2,215,691 USD $15,332.03 in 2023 $16,280.15 in 2025		

Abbreviations used: aSAH=aneurysmal subarachnoid hemorrhage; IV=intravenous.

## Data Availability

Data is provided within the manuscript.
